# ENGAGING OLDER ADULTS AND CAREGIVERS IN THE DEVELOPMENT OF A COUNTYWIDE DEMENTIA STRATEGIC PLAN

**DOI:** 10.1093/geroni/igae098.2587

**Published:** 2024-12-31

**Authors:** Mariana Reyes, Noel Barragan, Lisa Craypo, Liz Schwarte, Anne-Celine Jeffroy-Meynard, Tony Kuo

**Affiliations:** Los Angeles County Department of Public Health, Los Angeles, California, United States; Los Angeles County Department of Public Health, Los Angeles, California, United States; Ad Lucem Consulting, Kensington, California, United States; Ad Lucem Consulting, San Francisco, California, United States; Ad Lucem Consulting, Ad Lucem Consulting, California, United States; Los Angeles County Department of Public Health, Los Angeles, California, United States

## Abstract

In 2021, the Los Angeles County Department of Public Health launched the CDC-funded Los Angeles County BOLD Initiative (LA BOLD), the primary outcome of which is a strategic plan focused on improving dementia infrastructure and supports in Los Angeles County. To ensure that the plan aligns with concerns and priorities of older adults and caregivers impacted by dementia, LA BOLD partnered with Ad Lucem Consulting to conduct a series of 10 listening sessions with 92 participants to share their perspectives, experiences, and concerns related to dementia awareness and care between September and October 2022. To ensure inclusion of a diverse cross-section of community voices, sessions were held either virtually or in-person and conducted in English, Spanish, Korean, and English/Samoan. Sessions were held in strategic partnerships with multiple community organizations spanning the county’s Supervisorial Districts. Session topics were organized around the three strategic plan focus areas: Hypertension Prevention & Management, Early Detection, and Advance Care Planning. Overall, participants were appreciative of the opportunity to discuss dementia and related health issues. Key findings point to disparities in knowledge and accessible services (e.g., culturally appropriate messaging, inconsistent detection/referral, stigma, insufficient caregiver supports). A number of actionable recommendations emerging from the findings can be addressed in the strategic plan, offering valuable insights for policymakers and public health professionals on how to address this condition in the county and other communities. This presentation will highlight key findings and provide suggested solutions to facilitating equitable services/resources for dementia prevention and management.

